# Evidence-based of conjunctival COVID-19 positivity: An Italian experience: Gemelli Against COVID Group

**DOI:** 10.1177/1120672120976548

**Published:** 2021-11

**Authors:** Maria Cristina Savastano, Gloria Gambini, Alfonso Savastano, Benedetto Falsini, Umberto De Vico, Maurizio Sanguinetti, Paola Cattani, Simona Marchetti, Anna Rita Larici, Francesco Franceschi, Angelo Santoliquido, Rossana Moroni, Andrea Cambieri, Rocco Bellantone, Francesco Landi, Giovanni Scambia, Stanislao Rizzo

**Affiliations:** 1 Ophthalmology Unit, “Fondazione Policlinico Universitario A. Gemelli IRCCS”, Rome, Italy; 2 Catholic University “Sacro Cuore”, Rome, Italy; 3 Institute of Microbiology, Catholic University of “Sacro Cuore”, Rome, Italy; 4 Department of Laboratory and Infectious Science, “FondazionePoliclinico Universitario A. Gemelli IRCCS”, Rome, Lazio, Italy; 5 Department of Diagnostic and Laboratory Medicine, Institute of Microbiology, Catholic University of “Sacro Cuore”, Rome, Lazio, Italy; 6 Department of Radiology, “Fondazione Policlinico Universitario A. Gemelli IRCCS”, Rome, Lazio, Italy; 7 Emergency Medicine, “Fondazione Policlinico Universitario A. Gemelli IRCCS”, Rome, Lazio, Italy; 8 Scientific Direction, “Fondazione Policlinico Universitario A. Gemelli IRCCS”, Rome, Lazio, Italy; 9 Hospital Health Management, “Fondazione Policlinico Universitario A. Gemelli IRCCS”, Rome, Italy; 10 Division of Endocrine and Metabolic Surgery, “Fondazione Policlinico Universitario A. Gemelli IRCCS”, Rome, Lazio, Italy; 11 Department of Geriatrics, “Fondazione Policlinico Universitario A. Gemelli IRCCS”, Rome, Lazio, Italy; 12 “Dipartimento scienze della salute della donna e del bambino e di sanità pubblica”, “Fondazione Policlinico Universitario A. Gemelli IRCCS”, Rome, Lazio, Italy; 13 Consiglio Nazionale delle Ricerche, Istituto di Neuroscienze, Pisa, Italy

**Keywords:** Conjunctival swab, COVID-19, eye infection, ophthalmologists, precautionary measures, personalized medicine, SARS-CoV-2

## Abstract

**Background:**

The possible transmission of severe acute respiratory coronavirus 2 (SARS-CoV-2) by tears and conjunctiva is still debated.

**Methods:**

Main outcome was to investigate the agreement between nasopharyngeal swab (NPs) and conjunctival swabs (Cs) in patients with SARS-CoV-2 infection. We divided patients into four groups: (1) NPs and Cs both negative (C−NF−), (2) NPs positive and Cs negative (NFs+Cs−), (3) NPs negative and Cs positive (NFs−Cs+), and (4) NPs and Cs both positive (NFs−Cs+). The secondary outcomes were to correlate Cs results with systemic clinical parameters such as: oxygen saturation (SpO_2_), dyspnea degree (DP), radiologic pulmonary impairment based on chest radiography (XR) or computed tomography (CT), blood chemistry as D-Dimer (D-Dimer), fibrinogen, ferritin, lactate dehydrogenase (LDH), and C-reactive protein (C-RP).

**Results:**

A total of 100 conjunctival swabs in 50 patients with SARS-CoV-2 have been enrolled in this interventional clinical trials. Ocular signs (conjunctivitis) were present in five patients (10%). NPs and Cs highlighted a poor level of agreement (0.025; *p* = 0.404). Median SpO_2_ levels are the highest in the NF−C− group (98%) and the lowest (90%) in the group NF+C+ (*p* = 0.001). Pulmonary impairment was statistically significantly different between NFs and Cs groups (*p* = 0.019). Pulmonary impairment score increased from NFs−Cs− group (3.8 ± 3.9), to NFs+Cs+ group (6.7 ± 4.1). Intensive care unit patients showed higher COVID-19 Cs positivity in conjunctiva (12.5%) against hospitalized ones (5.8%).

**Conclusions:**

In patients hospitalized for SARS-CoV-2 the virus can be detected in conjunctival swab. Intensive care unit patients may reveal a higher COVID-19 presence in the conjunctiva. The most severe pulmonary impairment can be observed in NFs and Cs positivity.

**Trial registration:**

Clinicaltrials.gov registration.

**Ethical committee authorization:**

ID number: 0013008/20

## Introduction

In recent months, humankind has been in jeopardy. Significant worldwide alarm is spreading as a result of infection by Severe Acute Respiratory Syndrome-Coronavirus-2 (SARS-CoV-2) infection.^1^ Following the outbreak in China, Italy, northern region of Lombardy, has become one of the areas of the world with the highest incidence of SARS-CoV-2.^2^ As a result of this emergency, several aspects of human life have changed; quarantine has become mandatory in many countries from East to West. Today the only available parameter that seems effective is the early diagnostic screening through nasopharyngeal swab (NPs) followed by self-isolation or quarantine of affected asymptomatic/symptomatic patients. Nonetheless, recent data reports that detection rate via nasal swab is only 63% and via pharyngeal swab is slightly more than half at 32%.^3^

New methods of diagnosis based on transcriptome sequencing of the RNAs isolated from the bronchoalveolar lavage fluid and peripheral blood mononuclear cells of coronavirus disease 2019 (COVID-19) patients are being evaluated.^4^

While science continues to quickly progress in terms of new diagnostic and therapeutic answers aimed at personalized medicine, it becomes increasingly evident that some patients are paucisymptomatic, while others move inexorably towards decline and death. Although risk factors for poor outcome have been described including advanced age, male gender and presence of comorbidities (obesity, diabetes, heart disease, lung disease, kidney disease), it has been suggested that immunosuppressed patients are not at increased risk of severe complications compared to the general population.^5^ These findings suggested that COVID-19 infection may cause massive alterations of the host transcriptome, inducing an aberrant metabolism of the host cells. As a consequence of this aberration, an abnormal immune response become the consequently leads to an ideal viral replication. Concomitant venous thromboembolism (VTE) has been described as a potential cause of unexplained deaths, but its management was challenging due to the complexity between antithrombotic therapy and coagulation disorders.^6^

Although these new hypotheses enrich our knowledge of the action mechanism of the SARS-CoV-2, the number of victims is increasing, and unfortunately some of them are also very young. One well-known case is that of our colleague, the young Chinese Ophthalmologist Dr Li Wenliang, that was among the first having probably a flair for a possible pandemic. In memoriam of this 34-year old Colleague (February 7, 2020), it was reported that ophthalmologists can be more than eye doctors.^7^ In this memorial it was described how, first among all, Dr Li recognized a new type of eye disease in seven patients with suspect respiratory disease. Probably, he didn’t have time to completely describe his findings because, unknowingly, he was infected by the COVID-19 and shortly thereafter died. His story has spread around the world and inspired researchers to investigate the presence of the COVID-19 within the eye as in tears, conjunctival secretion, and conjunctival epithelial cells.^8^

The presence of the virus in tears is controversial. Xia et al.^9^ described the presence of COVID-19 in the tear film using real-time reverse transcription polymerase-chain reaction (RT-PCR) assays only in one patient with conjunctivitis among the individuals infected by SARS-CoV-2. Conversly Seah et al.^10^ described the low risk of ocular transmission, since neither viral culture nor reverse transcription polymerase chain reaction (RT-PCR) detected the virus in conjunctiva. Recently, an Italian research organization of Spallanzani Group, described a case of concomitant NPs negativity versus conjunctival swab positivity in a SARS-Co-V2 patient.^11^ This finding opens questions about correct diagnosis in clinical practice, highlighting the potential role of conjunctival testing. Similarly, the way of transmission via the conjunctiva and tears cannot to be excluded.

The main goal of our study was to investigate the positivity of the COVID-19 in the conjunctival mucosa, in order to evaluate a possible new diagnostic tool. The secondary goal was the correlation between the conjunctival positivity and the disease-related systemic impairment. Both these goals could be relevant for a personalized medicine.

## Methods

### Study Oversight

The interventional prospective trial was supported by Fondazione Policlinico A. Gemelli IRCSS Rome, Italy and designed by the investigators. It was approved by the Catholic University/Fondazione Policlinico Gemelli IRCCS Institutional Ethical Committee (protocol ID number: 0013008/20 authorized on March 20th, 2020). The study adhered to the declaration of Helsinki. Clinical trials registration was applied: ID number 0013008/20.

Informed consent was collected for each patient. A complete explanation of the target protocol was fully described. Data were analyzed and interpreted by the authors. All the authors reviewed the manuscript and vouch for the accuracy and completeness of the data and for the adherence of the study to the protocol, available with the full text of this article at NEJM.org.

### Data sources

The raw sequencing data as well as all patient investigations from this study have been deposited in Trackcare system of Fondazione Policlinico Universitario A. Gemelli IRCCS Data Center, Rome, Italy (https://www.policlinicogemelli.it/news-eventi/sistema-informativo-sanitario-il-gemelli-sceglie-la-soluzione-trakcare-di-intersystem/)

We obtained the medical records through the compiled data for hospitalized and intensive care unit (ICU) patients confirmed or extremely suspected for Covid-19 infection.

Data were collected from hospitalized patients from 26th March 2020 and 21th April at “Fondazione Policlinico A. Gemelli IRCSS” in Rome, Italy. Emergency, ICU, microbiology and ophthalmology medical doctors cooperated to merge all the information and to evaluate new targets of personalized medicine. All patients included in the study were admitted to our Italian hospital through the emergency department during coronavirus peak pandemy.^12^

One-hundred eyes of 50 SARS-CoV-2 patients were enrolled in for this interventional prospective study. Patients with laboratory-confirmed diagnosis through the NPs of SARS-CoV-2 and patients with SARS-CoV-2 pneumonia without NPs positive laboratory test were included in the study. On the same days, both conjunctival swab (Cs) and NPs were collected at the same time. The same laboratory performed the analysis for all swab types. We collected NFs, Cs, inflammation conjunctival signs, blood oxygen saturation (SPO_2_), mechanical ventilation necessity, dyspnea degree, cough presence and chest radiological findings, blood chemistry as D-Dimer (D-Dimer), fibrinogen, ferritin, lactate dehydrogenase (LDH), and C-reactive protein (C-RP). Data were analyzed in cooperation with microbiologists, internal medicine specialists, infectious disease consultants, radiology consultants, ophthalmologists, and emergency doctors to describe the precise correlation of each signs and symptoms.

### Ophthalmological sampling

**Figure 1.**
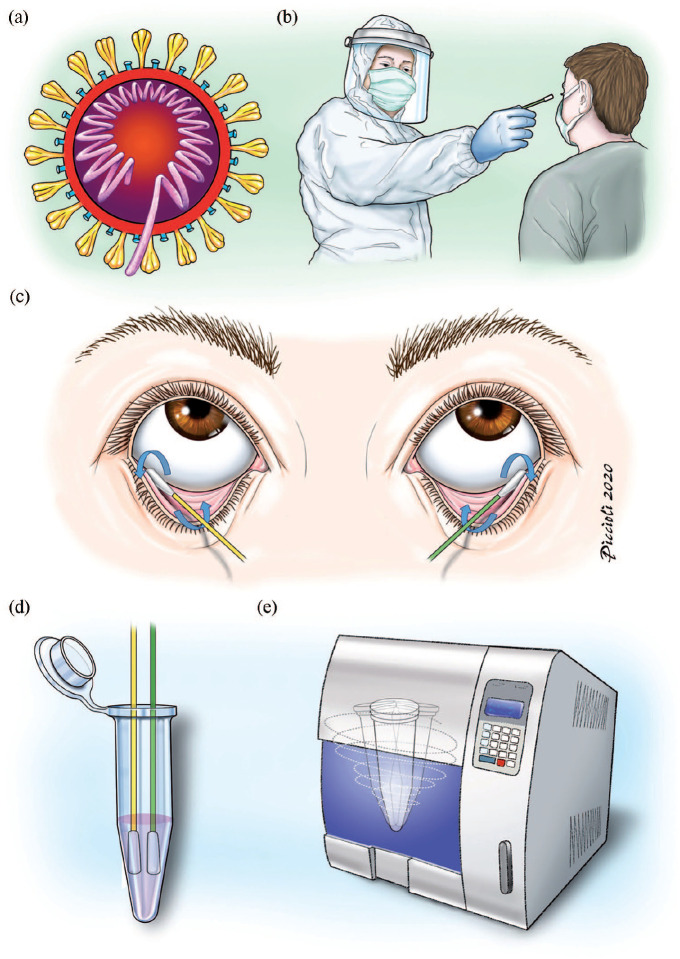
Shows the procedure of conjunctival swabs: (a) the virus COVID-19 containing single-stranded (positive-sense) RNA associated with a nucleoprotein within a capsid comprised of matrix protein, (b) conjunctival swabs performed in personal protection equipment, (c) swabs of both eyes were taken by rolling cotton swabs across the lower fornix conjunctiva of both eyes, (d) both swabs, one for each eye, were introduced in the same virus-specific tube and delivered to the laboratory for analysis (e).

Despite not performing any aerosol-generating maneuvers, the close proximity of patient contact could have increased the personal risk of exposure to COVID-19.^13^ Adequate personal protection equipment (PPE) was donned. Protective suit, glasses, double gloves and filtering face piece 2 (FFP2) mask were mandatory during the procedure of swabbing the eyes. To minimize the infection risk, we did not remain in the patient’s recovery room for more than 10 min to minimize the infection risk. As shown in Figure 1, rolling cotton swabs across the conjunctiva of the lower fornix was performed bilaterally. Both swabs, one for each eye, were inserted into the same tube containing virus-specific transport medium (UTM, Copan, Italy) and delivered to the laboratory for virological examination. All Cs were performed by only two ophthalmologists (SR and MCS).

### Microbiology procedure

Conjunctival swabs were processed on European Community-In Vitro Diagnostic Medical Device (EC-IVD) marked NIMBUS Automated Liquid Handling Workstations from nucleic acid (NA) Extraction to PCR Setup (Seegene, Arrow Diagnostics, South Korea).

SARS-CoV-2 RNA was detected by multiplex Real-time RT-PCR assay using Allplex™ 2019-nCoV Assay (Seegene, Arrow Diagnostics, South Korea) on CFX96 Real-time detection system (Biorad, Italy) according to the manufacturer’s directions.

Allplex™ 2019-nCoV Assay is a multiplex Real-time PCR assay for simultaneous detection of 3 target genes of SARS-CoV-2 in a single tube. The assay is designed to detect = RNA-dependent RNA polymerase (RdRP) and nucleocapsid protein gene (N-genes) specific for SARS-CoV-2, and envelope gene (E-gene) for all of Sarbecovirus including SARS-CoV-2. E gene and RNA-dependent RNA polymerase (RdRP) gene were recommended by German Center for Infection Research (DZIF), and N-gene was recommended by the US Centers for Disease Control and Prevention and the Chinese Center for Disease Control and Prevention. This test was approved for emergency use authorization from Korea Centers for Disease Control and Prevention and also CE-IVD marked.

Negative samples were re-tested and nucleic acids were extracted and enriched using the automated EZ1 Advanced XL system (Qiagen, Milano, Italy) with the EZ1/DSP Virus Kit (Qiagen). Extractions were performed following the manufacturer’s instructions. Extracted samples were then submitted to amplification reaction for viral RNA detection (Allplex™ 2019-nCoV Assay). Procedures to prevent specimen contamination and PCR carryover were rigorously observed at all stages.

### Radiologic and laboratory findings

To detect a possible correlation between conjunctival swab positivity and clinical demographic features, we collected the exposure history of each enrolled patient. Further clinical signs or symptoms and radiological assessment were also considered.

Radiologic assessments included chest radiography or computed tomography (CT), and we established the radiologic abnormality on the imaging evaluated by “two chest radiologists” who defined the degree of severity of SARS-CoV-2 as a score according to previous studies.^14,15^ Possible disagreement between two observers was resolved by consultation with a third reviewer to obtained a consensus.

### Statistical analysis

Sample size calculation was calculated considering a test for agreement between two raters using the Kappa statistic, a sample size of 50 subjects achieves 95.7% power to detect a true Kappa value of 0.80 in a test of H0: Kappa = κ0 versus H1: Kappa ≠ κ0 when there are two categories and significance level of 0.05.

The sample was described in its clinical and demographic characteristics applying descriptive statistics techniques. Categorical variables were described with absolute frequencies and percentage tables (*n*, %); continuous variables were summarized with mean and standard deviation. Normality of data was checked using Kolmogorov-Smirnov test. Results from both the swabs (conjunctival and nasopharyngeal) were compared to evaluate agreement. The following measures were calculated: sensitivity (along with 95% CI), specificity and Cohen’s Kappa index.

Patients were classified according to both swabs results as follows: NFs+Cs+ (patients positive for both the swabs), NFs+Cs− (patients positive only to nasopharyngeal swab), NFs−Cs− (patients negative to both the swabs), and NFs−Cs+ (patients negative to nasopharyngeal swab and positive to conjunctival swab). This classification has been named “Patient Status.” Kruskal-Wallis H test was performed to evaluate the difference between median SpO_2_ level in the different groups. Pairwise comparisons were performed using Dunn’s procedure with Bonferroni correction for multiple comparisons. One-way ANOVA was performed to evaluate whether there were any statistically significant differences among the means of the independent groups concerning pulmonary impairment. Statistical analysis has been performed with SPSS 25.

## Results

A total of 50 patients, 36 males (59%), and 14 females (23%) were enrolled in this prospective interventional study. Mean age was 69.6 years (±13.1), minimum age was 40 years, and maximum age was 95 years. A total of 44 patients (88%) tested positive for the NFs and only four patients (8%) tested positive to Cs. Ocular signs (conjunctivitis) were present in five patients (10%), all of whom tested positive to the nasopharyngeal swab and one of whom tested positive to both the swabs. Table 1 summarized clinical and demographic characteristics of the sample.

**Table 1.** Sample characteristics.

	No. of participants/**total no. (%)**
**Characteristic**	*N* = 50
**Male – sex**	36/50 (72.0)
**Age – year (Mean ± SD)**	69.6 ± 13.1
**Nasopharyngeal swab – positive**	44/50 (88.0)
**Conjunctival swab – positive**	4/50 (8.0)
**Ocular signs – positive**	5/50 (10.0)
**Patients status**	
**NFs+Cs+**	3/50 (6.0)
**NFs+Cs−**	41/50 (82.0)
**NFs−Cs−**	5/50 (10.0)
**NFs−Cs+**	1/50 (2.0)
**Mechanical ventilation – Yes**	8/50 (16.0)
**Cough – Yes**	22/50 (44.0)
**Dyspnea**	
**5**	31/50 (62.0)
**4**	4/50 (8.0)
**3**	1/50 (2.0)
**0**	7/50 (14.0)
**Not classified**	7/50 (14.0)
**SpO_2_ (**mean** ± SD)**	94.7 ± 4.6
**Temperature (**mean** ± SD)**	36.4 ± 0.8
**Pulmonary impairment score (**mean** ± SD)**	8.6.4 ± 4.4
**Days from first symptom**	17.8 ± 10.2
**D-Dimer**	3221.2 ± 5017.8
**Fibrinogen**	536.5 ± 214.8
**Ferritin**	753.5 ± 403.1
**LDH**	271.6 ± 98.4
**C-RP**	73.61 ± 84.4

Agreement among the two swabs results was evaluated. Sensitivity was 6.8% with 95% CI = (2–19), specificity was 83.3%. Cohen’s Kappa index highlighted a poor level of agreement (0.025; *p* = 0.404).

From our sample we had that three patients (6.0%) were positive to both the swabs (NFs+Cs+), five patients (10.0%) were negative to both the swabs (Cs−NFs−), 41 patients (82.0%) were positive only to NFs (NFs+Cs−), and 1 patient (2.0%) was negative to NFs and positive to the conjunctival one (NFs−Cs+). The only patient presenting positive conjunctival swab and negative nasopharyngeal swab has been considered for descriptive statistics but has been excluded from one-way ANOVA and Kruskal-Wallis.

**Figure 2.**
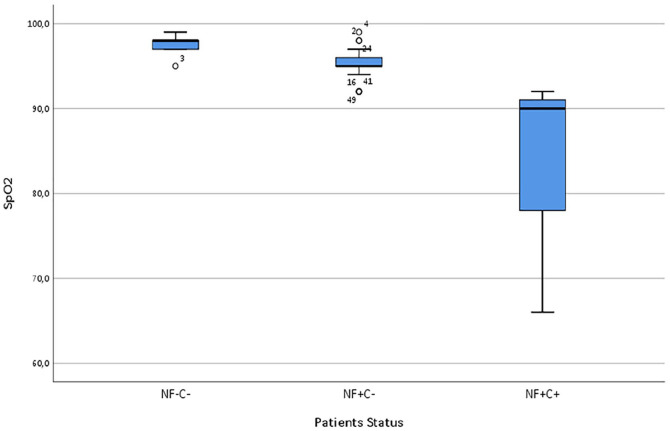
Box-plot shows the SpO_2_ (%) correlated to NFs−Cs patient status. Highest group was NFs−Cs− (98%) and the lowest (90%) was NFs+Cs+ group. The values were statistically significantly between groups H(2) = 13.062, *p* = 0.001; eta-squared = 0.482. Adjusted *p*-values was presented: (NFs−Cs−) versus (NFs+Cs+), *p* = 0.001; (NFs+Cs+) versus (NFs+Cs−), *p* = 0.024.

Median SpO_2_ levels was the highest in the (NFs−Cs−) group (98%) and the lowest (90%) for the group (NFs+Cs+). According to Kruskal-Wallis test, median SpO_2_ scores were statistically significantly different across groups H(2) = 13.062, *p* = 0.001; eta-squared = 0.482. Adjusted *p*-values was presented: (NFs−Cs−) versus (NFs+Cs+), *p* = 0.001; (NFs+Cs+) versus (NFs+Cs−), *p* = 0.024, see Figure 2.

**Figure 3.**
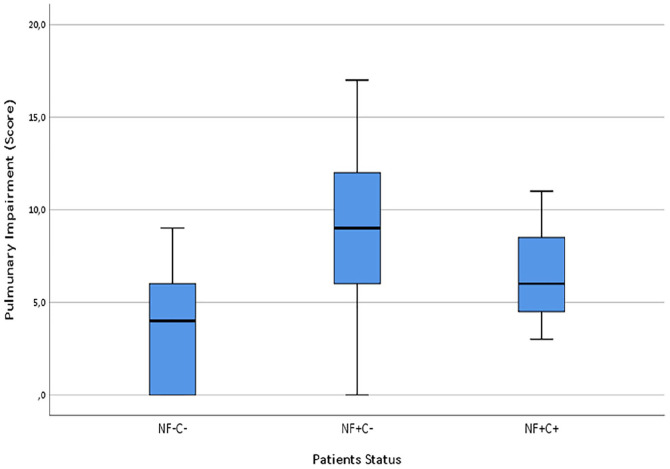
Box plot reports the pulmonary impairment (score) correlated to NFs and Cs patient status. Statistically significantly differences among different patients status groups, F(2, 46) = 4.330, *p* = 0.019 has been observed. Pulmonary impairment score increased from the (NFs−Cs−) group (3.8 ± 3.9), to (NFs+Cs+) group (6.7 ± 4.1), to (NFs+Cs−) group (9.4 ± 4.2). Tukey post hoc analysis revealed that the difference between (NFs−Cs−) and (NFs+Cs−) was statistically significant (*p* = 0.019; 95%CI = [−10.3; −0.78]).

Pulmonary impairment score increased from the (NFs−Cs−) group (3.8 ± 3.9), to (NFs+Cs+) group (6.7 ± 4.1), to (NFs+Cs−) group (9.4 ± 4.2). Tukey post hoc analysis revealed that the difference between (NFs−Cs−) and (NFs+Cs−) was statistically significant (*p* = 0.019; 95%CI = [−10.3; −0.78]). Figure 3

Patients who needed intensive care showed a higher COVID-19 Cs positivity in conjunctiva (12.5%) against the ones admitted in non-intensive care (5.8%). One-way ANOVA was conducted to determine if the pulmonary impairment score was different for patient’s status groups. Data was normally distributed for each group, as assessed by Shapiro-Wilk test (*p* > 0.05); and there was homogeneity of variances, as assessed by Levene’s test of homogeneity of variances (*p* = 0.876). Data was presented as mean ± standard deviation. Pulmonary impairment score was statistically significantly different between different patients status groups, F(2, 46) = 4.330, *p* = 0.019.

No other between-group differences were statistically significant for D-dimer, fibrinogen, ferritin, LDH, and C-RP.

## Discussion

The infection of COVID-19 virus is responsible for severe acute respiratory syndrome-related coronaviruses (SARS-CoV2) with significant morbidities and mortalities around the world. Coronavirus infection by another subtype of virus was previously described during the SARS-CoV epidemics in 2006.^16^ The authors reported how the ophthalmologists may be particularly susceptible to the infection during routine ophthalmic examinations by direct ophthalmoscopy and slit-lamp examination. The primary route of transmission of all SARS-CoV appears to involve close person-to-person contact through droplets. As recently reported, the COVID-19 virus transmission through the ocular surface should not be ignored.^8,17^

To date, no diagnostic tools have demonstrated efficacy for patients with Covid-19 although the oro/nasopharyngeal swab is being considered as the most adopted diagnostic approach. Wang et al.^3^ reported that the pharyngeal swab was diagnostically effective only in 32% and nasal swab was able to detect only the 63% of the cases,^4^ implying that many false negative may still be present in the world. Xi et al.^9^ tested the conjunctival samples for SARS-CoV-2 RNA in 30 infected patients, and the conjunctival samples from one patient were positive for the virus on 3 days afterward. On the contrary, Seah et al.^10^ observed that there was no evidence of SARS-CoV-2 shedding in tears through the course of the disease, suggesting a low risk of ocular transmission.

These findings did not exclude the possible transmission of COVID-19 through the droplets of SARS-Co-V2 infected patients to the conjunctiva of a healthy patient.

The absence of univocal data in the current literature of the possible role of tears or conjunctival presence of virus, requires a more detailed correlation with the degree of systemic disease severity.

Our report describes the conjunctival COVID-19 virus presence in a few cases (8%).

According to Chen et al.,^18^ conjunctival sampling might not be useful for early diagnosis because the virus may not appear initially in the conjunctiva. However, as described in our study, Colavita et al.^11^ reported a case of conjunctival swab positivity and concomitant NFs negativity suggesting a new potential of false negative patients.

Based on the current results, we may speculate that the presence of COVID-19 in the conjunctiva may be related to the severity of pathology. Our findings showed that NFs and Cs were both positive in patients with the most severe systemic impairment. Although we do not have knowledge of the detailed mechanism underlying this correlation, we hypothesize an increased systemic as well as conjunctival viral load for patients who are positive for both tests.

The almost complete absence of conjunctival inflammatory reaction in Cs+ (only one case of conjunctivitis in Cs+) implies good conjunctival tolerability in the presence of the virus, probably linked to a low local antigenic reactivity. In our knowledge this study first of all reported a high percentage of Cs positivity in patients with no signs of conjunctivitis. Yet, it is difficult to interpret this finding, since in general the virus infection in the conjunctiva should induce a marked local hyperemia. A possible explanation could be related to the COVID-19 behavior of hiding very effectively into the cells of the conjunctiva, avoiding any minimum protein dispersion without being recognized. This occurrence would also explain the almost total absence of the virus in the film tears as demonstrated by Seah et al.^10^

In our results, intensive care unit patients showed higher COVID-19 Cs positivity in conjunctiva (12.5%), suggesting that disinfection of the conjunctival should also be considered.

Currently, our understanding of the possible ocular complications of SARS-CoV-2 infection is very limited. Probably in the near future we will see different ocular tissue involvement secondary to COVID-19 infection.

Interpretation of the results of our study is limited by the absence of duration of follow-up, the lack of a healed patients group.

As we wait for new diagnostic targets to allow us a more accurate diagnosis for personalized therapy, conjunctival swab as well oro-pharyngeal swab or other new emerging hematological tests,^19,20^ currently do not appear to be sufficiently reliable in the clinical practice.

In conclusion, our findings suggest that the way of contagion through the eyes is possible although with low risk from positive patients, except for cases with systemic deterioration related to SARS-CoV-2 consequence. Combined positivity of both tests is suggestive of more severe pulmonary involvement with respiratory failure.
